# Automated opportunistic cardiovascular risk assessment in non-small cell lung cancer patients on routine chest CT using an optimised nnU-net framework

**DOI:** 10.1186/s12880-026-02252-z

**Published:** 2026-03-03

**Authors:** Jubril Olayinka Anifowose, Zechen Li, Girija Agarwal, Eric O. Aboagye, Declan P. O’Regan, Ben Ariff, Susan J. Copley, Mitchell Chen

**Affiliations:** 1 Department of Medicine, Mid and South Essex NHS Foundation Trust, Prittlewell Chase, Westcliff-on-Sea, Southend-on-Sea, SS0 0RY UK; 2 Department of Surgery and Cancer, Imperial College, Du Cane Road, London, W12 0NN UK; 3 Imperial College Healthcare NHS Trust, Du Cane Road, London, W12 0NS UK; 4 MRC Laboratory of Medical Sciences, London, W12 0HS UK; 5 Institute of Clinical Sciences, Imperial College London, London, W12 0HS UK

**Keywords:** Artificial intelligence, Cardiovascular disease, Lung cancer, Computed tomography, Opportunistic screening

## Abstract

**Background:**

Cardiovascular disease (CVD) and non-small cell lung cancer (NSCLC) are the global leading causes of overall and cancer-related deaths, respectively. NSCLC patients have a higher CVD risk than the general population which is frequently underdiagnosed. Coronary artery calcification (CAC), a marker of CVD, is commonly detected on routinely acquired CT from NSCLC work-up but often not reported. We present an automated CAC assessment tool validated for NSCLC patients using a deep learning-based framework to provide a non-invasive CVD screening opportunity without incurring extra workload or radiation exposure.

**Methods:**

We trained nnU-Net models on ungated, unenhanced chest CTs (*n* = 97) from Stanford AIMI dataset, and tested them on three mutually independent datasets: (1) ungated unenhanced CTs from AIMI (*n* = 95), (2) attenuation correction CTs from PET-CT scans of NSCLC patients at our institution (ICHNT, *n* = 87; age 67.8 ± 10.1 years; M:F 174:113), and (3) CAC-negative scans from TCIA (*n* = 50); and used the best performing model to produce CAC segmentations, post-processed with TotalSegmentator, to stratify patients into CVD risk groups, informing the need for dedicated cardiac clinic assessment.

**Results:**

For a CAC threshold of 100, the model achieved accuracy: 83.6%, sensitivity: 91.9%, specificity: 70.8%, positive predictive value (PPV): 82.9%, negative predictive value (NPV): 85.1%, F1-score: 0.87, kappa coefficient: 0.65 and Area Under the Receiver Operating Characteristic Curve (AUC) score of 0.899. For a threshold of 400, accuracy: 84.5%, sensitivity: 90.9%, specificity: 79.5%, PPV: 77.6%, NPV: 91.8%, F1-score: 0.84, and kappa coefficient: 0.69 as well as an AUC of 0.926.

**Conclusion:**

Our optimised deep learning model can benefit NSCLC patients by providing CVD risk information from their routine CT scans which may not acted upon otherwise, thus enabling a practical opportunistic screening solution for these patients.

**Supplementary Information:**

The online version contains supplementary material available at 10.1186/s12880-026-02252-z.

## Background

Cardiovascular disease (CVD) is the leading cause of overall patient mortality globally, accounting for approximately 32% of all deaths, or 19.8 million lives lost annually [1]. Non-small cell lung cancer (NSCLC) is, on the other hand, the world's commonest cause of cancer-related mortality [2]. NSCLC patients have a markedly increased early risk of CVD, driven by factors including advanced age, smoking history, systemic inflammation, and exposure to cardiotoxic therapies. The risk of CVD is 2.2 times higher in NSCLC patients than in the general population in the first six months of their diagnosis [3] but often goes undetected. Coronary artery calcification (CAC); a manifestation of atherosclerotic disease and an independent risk factor for CVD [4], has been shown to be present in 69% of thoracic CT scans of NSCLC patients [5].

Additionally, a meta-analysis showed that CAC is present on 52% of non-gated thoracic CT scans performed for non-cardiac indications, but only 57% of such scans were reported as being CAC positive [6]. Another study showed that 87.4% of non-dedicated CT scans did not include CAC in the report when it is present [7], highlighting a missed opportunity to its detection and timely management. The British Society of Cardiovascular Imaging/British Society of Cardiac Computed Tomography (BSCI/BSCCT) and British Society of Thoracic Imaging (BSTI) recommend the visual assessment of coronary calcification on ungated CT imaging with gross estimation of severity and a recommendation for optimisation of CVD risk factors [7]. Given a significant number of scans with CAC are not being reported as having CAC, this poses a risk to patients who would otherwise benefit from early optimisation of CVD risk factors and pharmacological prophylaxis but are being missed in their diagnosis in substantial numbers, which may potentially lead to the occurrence of otherwise preventable major adverse cardiovascular events (MACE) such as myocardial infarction [8].

In current clinical practice, most NSCLC patients would receive ^18^F-fluorodeoxyglucose positron emission tomography/computed tomography ([^18^F]FDG PET/CT) scan as part of their disease work-up and staging. These routinely acquired imaging data include the coronary arteries, providing a pragmatic opportunity for opportunistic coronary artery calcification (CAC) assessment. The unenhanced nature of the attenuation correction CT of [^18^F]FDG PET/CT makes them amenable to opportunistic screening for CAC, a topic not yet explored in literature for this patient group but is invariably important in the context of CVD detection in NSCLC patients. CAC is normally assessed on dedicated non-enhanced cardiac CT with ECG-gating, but previous studies have shown a high level of concordance between CAC scores on dedicated and non-dedicated scans [9]. Numerous automated CAC scoring models have been developed, with some showing promising results in assessing CVD risk by automatically segmenting and quantifying CAC on thoracic and cardiac CT scans [10, 11]. However, many of these models were trained and validated on ECG-gated CT images [11], and of the small number that were intended for use with non-ECG-gated CT, they were not tested on NSCLC patients to support clinically meaningful implementation [10, 12, 13].

**Fig. 1:**
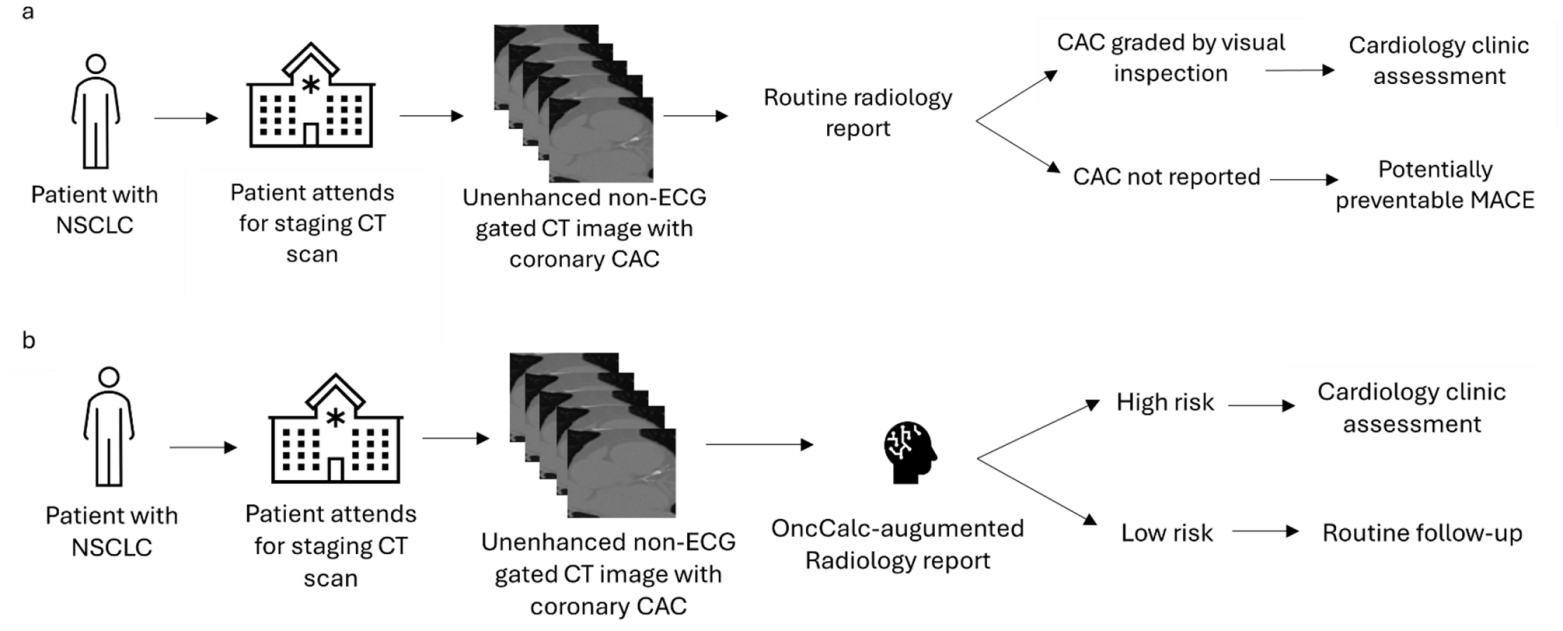
The proposed two-step CVD triaging process for NSCLC patients powered by OncCalc. **a**) Current standard practice for routine cancer workup and staging. **b**) Proposed OncCalc-assisted detection of CAC in this patient cohort. In this paradigm, NSCLC patients who have a high CAC evident on their CT imaging but not reported formally by radiology would be safety-netted by OncCalc

In this paper, we present an AI tool, *OncCalc*, to opportunistically detect CAC in NSCLC patients, as a prompt for appropriate cardiology clinic assessment using information from routine staging scans. *OncCalc* is capable of automatically assessing CVD risk in NSCLC patients on their routinely acquired, non-ECG gated cancer CT scans to stratify them into a low-risk group: where no further treatment/investigation is necessary, and a high-risk group: where appropriate cardiology assessment is prompted. This approach has the benefits of being automated which requires with no extra radiology work, such that all NSCLC patients who have had a CT scan covering their heart may be automatically assessed for CAC and referred to or followed up appropriately with cardiology (Fig. 1). The key novelties with OncCalc are its target population of NSCLC patients, and public domain availability to support an end-to-end usage. A web-based testing platform is in development, to further increase its outreach and accessibility.

## Materials and methods

This is a retrospective observational study on the development and testing of an AI tool based on patient data, adhering to the STROBE and TRIPOD-AI guidelines [14]. The study was approved by the institutional review board and Health Research Authority UK (HRA 18HH4616), and conducted in accordance with the Declaration of Helsinki. The requirement for informed consent was waived due to the study’s retrospective and observational nature and use of de-identified patient data.

### Study cohorts

For model training, we included unenhanced ungated staging chest CT scans of NSCLC patients from the Stanford University Centre for Artificial Intelligence for Medicine (*AIMI-Training*, *n* = 97) dataset, *AIMI-Training* [15]. For model testing we used three mutually independent data sets: (1) Testing set 1 (*AIMI-Testing*, *n* = 95): non-enhanced gated CT scans from AIMI (*n* = 95) [15]. (2) Testing set 2 from Imperial College Healthcare NHS Trust (*ICHNT*, *n* = 87): consecutive unenhanced ungated attenuation correction CT of the staging PET-CT scans from patients diagnosed of NSCLC at our multi-centre institution, as part of a retrospective observational study (HRA: 18HH4616)(Table 1). Six cases were excluded due to data corruption. (3) Testing set 3 (*TCIA*): CT scans with no CAC (*n* = 50) from The Cancer Imaging Archive (TCIA): acquired from COVID-19 patients without NSCLC [16]. The aim of the TCIA testing arm is to evaluate the model’s negative predictive value (NPV) performance in a more general, non-NSCLC population.

The ICHNT cohort data was acquired on Siemens Biograph 64 (Siements Healthcare, Erlangen, Germany). The PRT/CT scans were performed from upper thighs to the base of the skull following ≥ 4–6-h fasting and had a measured blood glucose level < 11.0mmol/l at the time of injection. A non-contrast CT scan (80-140 mA, 100–140 kVp) was conducted for both attenuation correction of PET data and co-registration with PET images. The imaging protocol and scanner makes in the AIMI and TCIA datasets were not publicly available.

The training and testing datasets were selected to maximise both representativeness and diversity within the NSCLC population, ensuring coverage of a range of CAC burdens, patient ages, and imaging acquisition parameters typical of routine staging scans. Dataset sizes were determined based on prior work demonstrating adequate sample sizes for robust nnU-Net model training while balancing the availability of relevant imaging data. AIMI-Training was split 80:20 into training and internal validation, to maximise the amount of data available for model learning while reserving a sufficient subset to monitor performance, tune hyperparameters, and detect potential overfitting during training. This split is consistent with common practice in medical imaging studies, balancing robust model training with reliable internal validation.

CONSORT diagrams for these datasets can be found as Figure S1 in the supplementary material. A summary of the study datasets is presented in Table 2.

**Table 1:** Patient demographics of the ICNHT cohort. Performance scores are based on the Eastern Cooperative Oncology Group (ECOG) system and NSCLC staging based on International Association for the Study of Lung Cancer (IASLC) 8th edition. Patient demographics of AIMI and TCIA cohorts were not disclosed by their respective sources. Non-small cell lung cancer (NSCLC), Tumour stage (T stage), Nodal stage: (N stage), Metastasis stage: (M. stage)

Patient Characteristic	Number of Patients	
Age (Mean ± SD)	68.0 ± 10.3	
Age range	[37,87]	
Sex	Male	52
	Female	34
	unknown	1
Ethnicity	Caucasian	50
	African/Afro-Caribbean	5
	Asian	6
	Other	25
	Unknown	1
Histology	Squamous cell	0
	Adenocarcinoma	20
	NSCLC unspecified	64
	Unknown	3
Performance score	0	42
	1	26
	2	12
	3	6
	4	0
	Unknown	1
NSCLC T stage	1a	9
	1b	16
	1c	1
	2	1
	2a	25
	2b	6
	3	14
	4	14
	Unknown	1
NSCLC N stage	0	43
	1	7
	2	21
	3	15
	Unknown	1
NSCLC M stage	0	67
	1a	6
	1b	12
	1c	0
	Unknown	2

**Table 2:** Summary of study cohorts. Artificial Intelligence for Medicine (AIMI), Imperial College Healthcare NHS Trust (ICHNT), The Cancer Imaging Archive (TCIA)

Characteristic	Training	Testing		
Dataset	AIMI-Training	AIMI-Testing	ICHNT	TCIA
Size	97	95	87	50
Training/validation split	80% training 20% internal validation	N/A	N/A	N/A
Protocol	Unenhanced ungated	Unenhanced gated	Unenhanced ungated	Unenhanced ungated

**Fig. 2:**
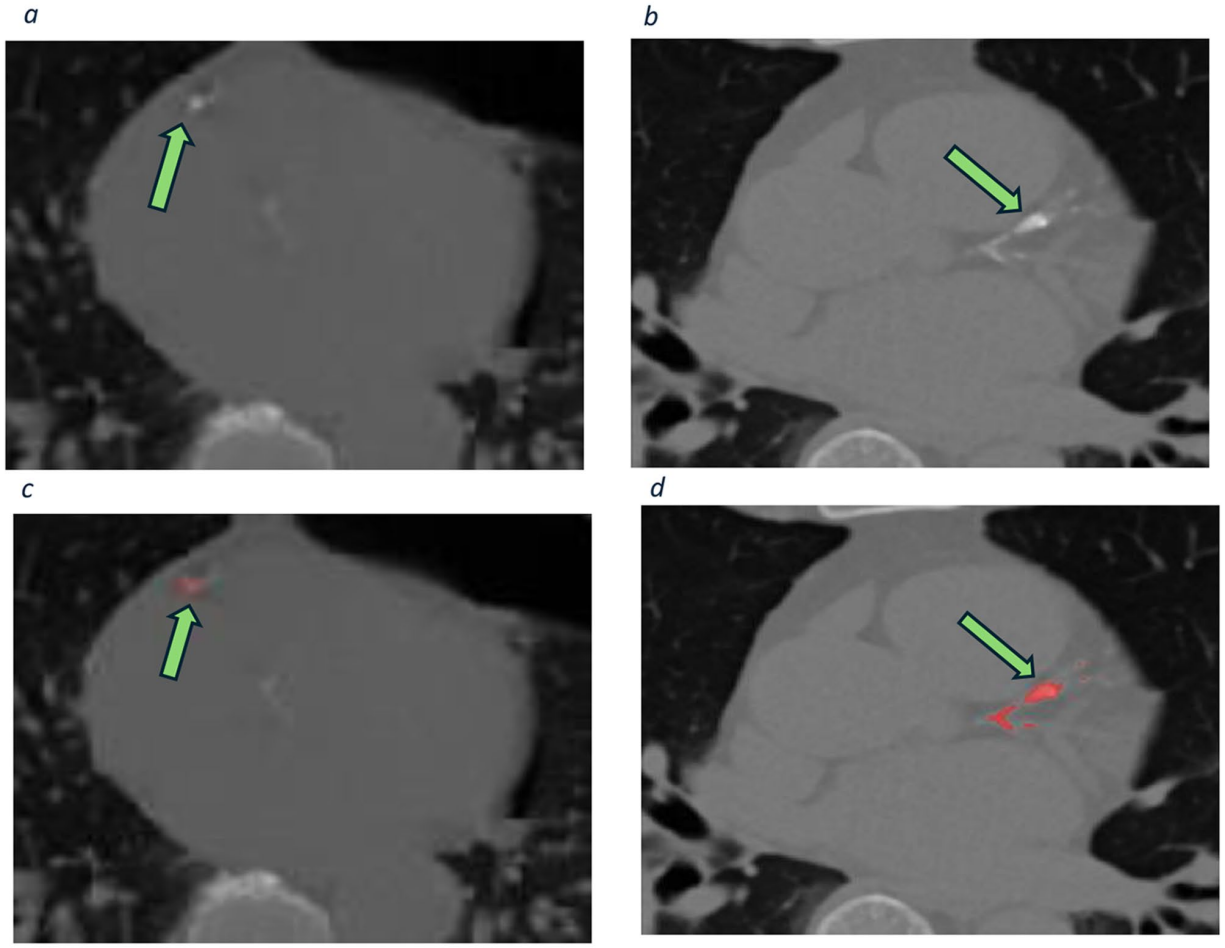
Coronary calcifications in two patients. The top row figures show CT scans with calcified areas in the coronary arteries. The bottom row figures are from the same studies with segmentation mask overlay. **a** & **c**) right coronary artery calcification; **b** & **d**) Calcifications in the left main stem, left anterior descending and left circumflex arteries. The regions of interests (ROI) are shown in red and additionally indicated by green arrows

To establish the ground truths, two clinical radiologists (MC and GA) of 9 and 2 years of professional standing, blinded to clinical data, double reviewed all scans and manually delineated calcifications (Fig. 2) in the coronary arteries (left main stem, left anterior descending, left circumflex and right coronary arteries), using 3D Slicer [17] (Slicer Community, Boston, USA). Case-wise complete inter-observer agreement was 95%, with a mean inter-observer variability of 0.87 (intra-class coefficient).

### Model development

**Fig. 3:**
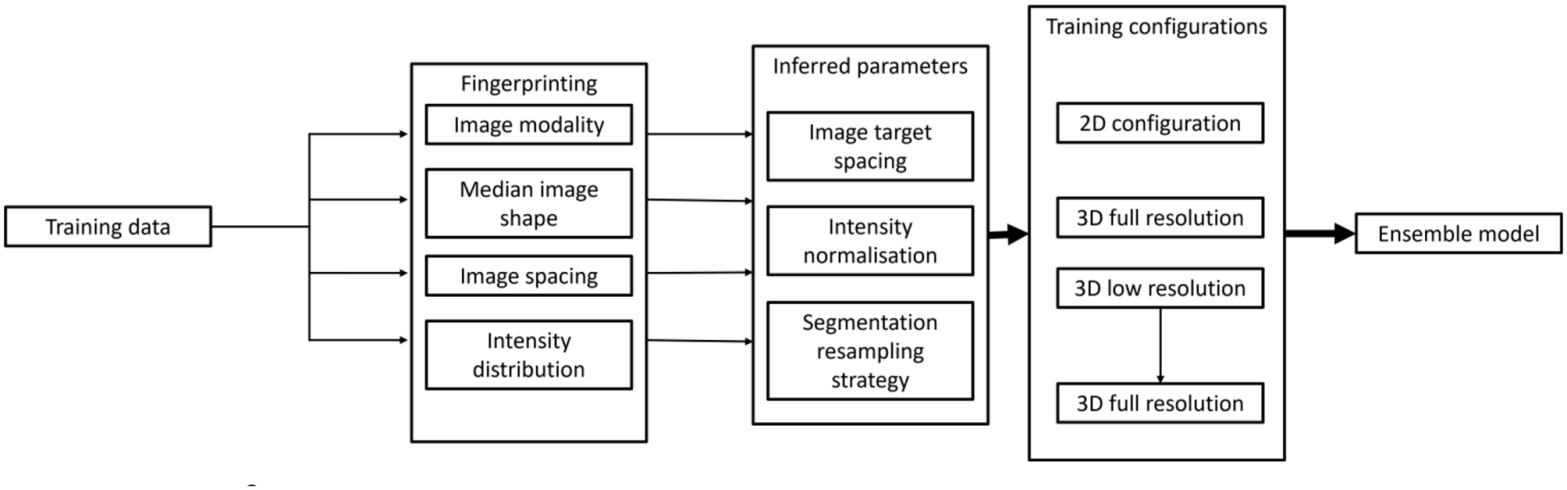
Self-configuring development pipeline of the nnU-Net model and testing

We developed an automated calcification quantification tool, *OncCalc*, which incorporates automated CAC assessment using a trained nnU-Net2 model [18], with CAC score computation and patient stratification. The AI tool is intended for use on unenhanced chest CT without ECG gating. The nnU-Net model development pipeline and overall OncCalc workflow are shown in Figs. 1 and 3, respectively.

We performed our model development and testing on a workstation with AMD Ryzen Threadripper PRO 5975WX 3.4 GHz central processing unit (CPU), 128GB system random access memory (RAM), and dual Nvidia GeForce RTX 3090 graphical processing units (GPU) with 24GB video RAM (VRAM) each.

nnU-Net is a deep learning architecture for well-known for its ability to automatically adapt to new medical image segmentation tasks with minimal tuning [18]. Its strong, out-of-the-box performance has been demonstrated in various imaging AI grand challenges and high impact publications. nnU-Net2 further builds on its predecessor with residual encoder U-net architecture which improve segmentation performance as well as improved ease of model hyperparameter tuning with the ‘plans’ files [18, 19].

nnU-Net model training calibrates hyperparameters such as patch and batch size according to the median image dimensions [18]. They are then optimised to generate a model which produces feature maps of size 8 × 8 × 8 pixels in six or fewer pooling operations for the specific image modality and size of the image dataset of interest whilst complying to hardware constraint such as available VRAM [18].

The training images are first pre-processed to generate a unique configuration profile, with *3d_fullress*, *3d_lowres*, *3d_fullcascade*, and *2d* configurations trained independently for 1000 epochs each, as shown in (Fig. 3), which was determined based on loss function monitoring. An ensemble of model configurations was then developed which combines independently trained configurations at various folds to improve overall performance and robustness by leveraging the strengths of the combined configurations. The best performing configuration was selected based on the average Dice score in the external testing datasets.

We trained and externally validated 3d full resolution with the recommended residual encoder settings to determine the best model configuration [19]. Ultimately, we focused on training with the *3d_fullres* configuration as it performed the best according to Dice score after the initial round of training all configurations.

For hyperparameter tuning, we experimented with batch sizes of 2,4,6,8,10 and 16 images, and patch sizes of 24 × 32 × 32, 24 × 64 × 64, 24 × 128 × 128 and 24 × 224 × 224 voxels. We did this because although nnU-Net self-configures the batch and patch size based on median image size and available VRAM, they are optimised to minimise memory requirements and training time whilst maintaining model performance which does not imply optimisation for a specific segmentation task [18].

We fine-tuned these hyperparameters because of the dual GPU setup of our workstation, and that the batch size must be divisible by the number of GPUs [18], as well as the total available VRAM of 48GB across the two GPUs. Lastly, we used the combination of batch size of 8 and a patch size of 24 × 128 × 128 as this performed the best out all trained configuration combinations.

### Post-processing

To improve the performance of coronary calcification segmentation, we carried out post-processing with TotalSegmentator [20]. The purpose is to exclude mis-labelled calcifications which can be found in other cardio-vascular structures such as the ascending aorta, arch of the aorta and descending aorta. TotalSegmentator can segment the chambers of the heart chambers and aorta, but not the coronary vessels (Figure S2 in the supplementary data).

### Model performance assessment

#### CAC scoring

CAC score quantifies coronary calcifications on CT [21] and thereby provides an estimate of the patient’s CVD risk for guiding subsequent lifestyle +/- pharmacological interventions [21, 22]. We calculate CAC scores in this study using the Agatston scoring system [6] and graded a CAC score of 0 as no identifiable disease, 1–99 mild atherosclerotic burden, 100–399 moderate atherosclerotic burden, 400 and above severe disease [22].

Clinical practise guidelines including those by the American College of Cardiology /American Heart Association (ACC/AHA), Canadian Cardiovascular Society (CCS), Cardiac Society of Australia and New Zealand (CSANZ) and the European Society of Cardiology (ESC) have differing criteria for up- or down-risking patients who have intermediate risk of CVD according to classical CVD risk factors. These guidelines also defined different thresholds for initiating statin treatment in this patient group. However, they all agree that a CAC score of 100 or above warrants, as a minimum; consideration of statin treatment, and a CAC score of 400 or above, definite initiation of statin [22].

We calculated the CAC scores for ground truth and predicted segmentations using an in-house software, then assigned them a binary “correctness score” of ‘0’ or ‘1’, based on whether they correctly stratified the patient to receive the correct subsequent management.

Given that the common theme among the clinical guidelines globally involves the consideration of statin therapy in cases of intermediate CVD risk with a CAC score of 100 and above [22], and to start immediate statin therapy in CAC scores of 400 and above [22]. We performed analysis using both 100 and 400 as thresholds to classify patients as high CVD risk therefore signalling them for further clinical CVD risk assessment. Following this, we calculated performance metrics including sensitivity, specificity, negative predictive value and positive predictive value for each model.

We performed analysis with both cutoffs as this may help to streamline the process of triaging as patients who are identified as having a severe coronary calcium burden/score ≥ 400 and might benefit from immediate statin treatment, as stated by the global clinical guidelines, prior to cardiology clinic assessment, compared to patients with calcium scores ranging from 100 to 400 [22]. Ultimately, all patients determined as having high risk/score ≥ 100 will be referred for cardiology clinic assessment to determine their suitability for dedicated coronary imaging.

Additionally, the F1-score was used as the performance metric of our model [23], and kappa coefficient as a measure of the agreement of methods [24].

All statistical analyses were performed using Python 3.11 (Python Software Foundation, Wilmington, USA), with packages *Scikit-learn 1.7.0*, *pandas 2.2.3* and *NumPy 2.1.3*. The statistical tests were two-sided, with a p-value threshold of significance at 5% adopted throughout.

## Results

We found the best performing nnU-Net model had a batch size of 8 and patch size of 24 × 128 × 128, which was selected for testing.

**Fig. 4:**
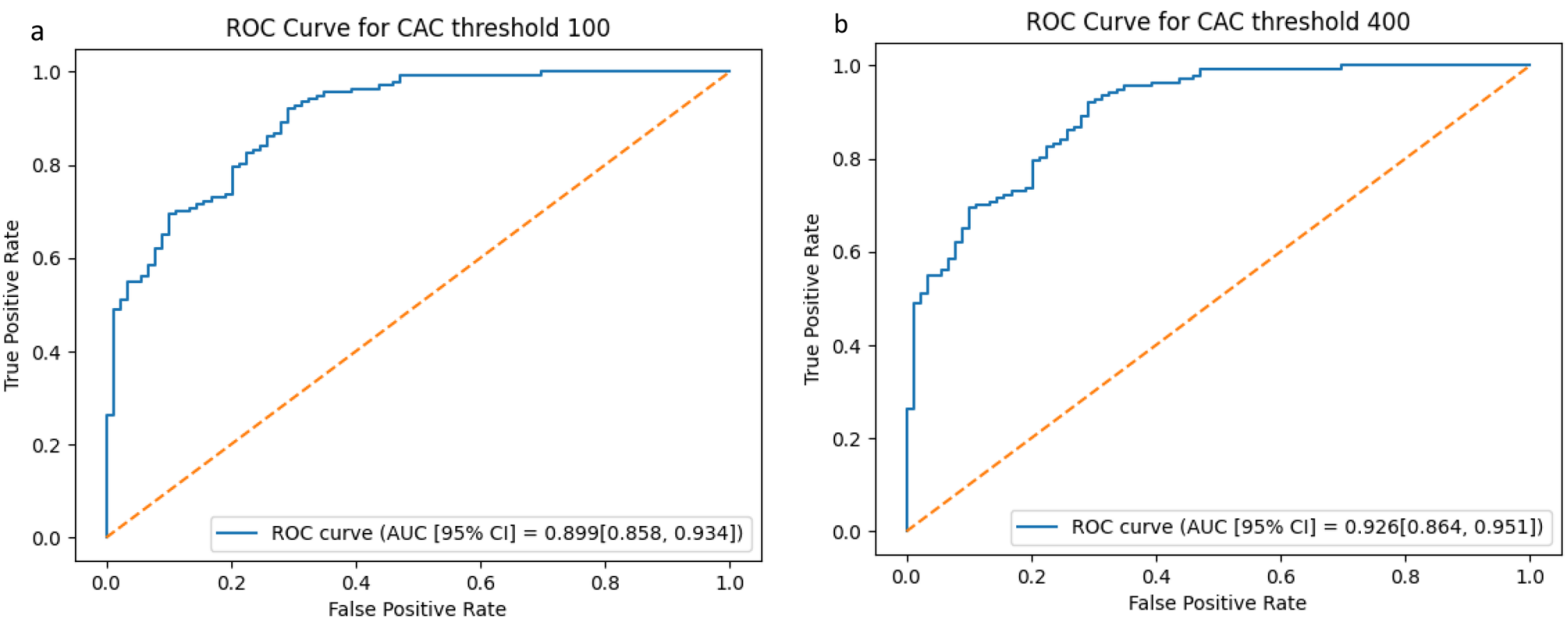
Receiver-operating characteristic (ROC) curves and area under the curve (AUC) analysis of *OncCalc*’s performance based on a CAC threshold of **a**) 100 and **b**) 400. Note good model performance in both cases.

*OncCalc* produced CAC scores that correctly guide clinical decision in (83.6%) 189 of 226 patients, in the external testing set (Table 3), using a threshold of 100, and 191 out of 226 (84.5%) patients using a threshold CAC of 400. With a CAC threshold of 100, the sensitivity of the model (Table 3) for risk stratifying patients to the high-risk group is 91.9%, specificity of 70.8%, positive predictive value (PPV) of 82.9%, negative predictive value (NPV) of 85.1%, F1-score [95% confidence interval] of 0.87 [0.83, 0.91] and kappa coefficient [95% confidence interval] of 0.65 [0.54, 0.75]. For a CAC threshold of 400, the model achieved a sensitivity of 90.9%, specificity of 79.5%, PPV of 77.6%, NPV of 91.8%, F1-score [95% confidence interval] of 0.84 [0.79, 0.91] and kappa coefficient [95% confidence interval] of 0.69 [0.56, 0.77]. Model performance on subclasses of the external testing set are presented in Table 4. In receiver-operating characteristic curve (ROC) analysis, the areas under the curve (AUC) [95% confidence interval] were 0.899 [0.858, 0.934] and 0.926 [0.864, 0.951] for CAC thresholds of 100 and 400, respectively (Fig. 4). Without TotalSegmentator post-processing, the model had accuracies of 83% and 80%, and kappa coefficients [95% confidence interval] of 0.62 [0.51, 0.72] and 0.61 [0.51, 0.71], for CAC thresholds of 100 and 400, respectively.

**Table 3:** Confusion matrices of model performance on combined dataset of all three external testing datasets: AIMI-COCA testing dataset, ICHNT and TCIA datasets. with CAC thresholds of 100 (top) and 400 (bottom). Artificial Intelligence for Medicine - Coronary Calcium and Chest CT (AIMI-COCA), Imperial College Healthcare NHS Trust (ICHNT), The Cancer Imaging Archive (TCIA)

Scores for combined dataset using CAC Threshold: 100		CAC scores based on ground truth segmentation	
		>=100	< 100
CAC score based on *OncCalc* segmentation	**>=100**	**126 (TP)**	**26 (FP)**
	**< 100**	**11 (FN)**	**63 (TN)**
Scores for combined dataset using CAC Threshold: 400		**CAC scores based on ground truth segmentation**	
		**>=400**	**< 400**
CAC score based on *OncCalc* segmentation	**>=400**	**90 (TP)**	**26 (FP)**
	**< 400**	**9 (FN)**	**101 (TN)**

TP= True positive, FP= False Positive, FN= False Negative, TN= True Negative

TP= True positive, FP= False Positive, FN= False Negative, TN= True Negative

**Table 4:** Table showing the sensitivity and specificity of the model for the different testing cohort as well as the composite dataset combining all three datasets including the TCIA to assess the model on a negative control dataset. N/A: not applicable in the TCIA cohort (all negative cases). Artificial Intelligence for Medicine - Coronary Calcium and Chest CT (AIMI-COCA), Imperial College Healthcare NHS Trust (ICHNT), The Cancer Imaging Archive (TCIA)

Performance measure	TCIA (*n* = 50)		AIMI-COCA (*n* = 95)		ICHNT (*n* = 81)		Combined Testing Cohort (*n* = 226)	
CAC Threshold	100	400	100	400	100	400	100	400
Sensitivity	N/A	N/A	0.82	0.72	1.0	1.0	0.92	0.91
Specificity	0.64	0.78	0.91	0.95	0.0	0.2	0.71	0.79
PPV	N/A	N/A	0.94	0.88	0.94	0.84	0.82	0.78
NPV	1.0	1.0	0.74	0.86	N/A	1.0	0.85	0.92

## Discussion

CVD and NSCLC are important disease entities known for their significant impact on patient health globally. The heightened risk of CVD in NSCLC patients is well recognised [3] but often underdiagnosed. The presence of CAC is normally assessed on dedicated cardiac imaging, a non-contrast enhanced ECG-gated CT scan where its presence is detected and quantified using the Agaston scoring system, to produce a risk metric which translates to a percentage of 10-year CVD risk to guide subsequent lifestyle modification and/or pharmacological intervention [25]. NSCLC patients routinely receive FDG-PET-CT scans as part of their disease work-up and staging, which includes an attenuation correction CT component, offering a route for CAC detection but is often missed in routine reporting practice [5–7]. To address this challenge, we developed an automated AI tool, *OncCalc*, for the opportunistic assessment of CAC on these images to safety-net NSCLC patients who may have high CVD risk manifesting as a high CAC burden. We tested the intended clinical use of OncCalc as a stratification tool to guide the need for dedicated cardiac assessment, in three mutually independent multi-national patient cohorts.

*OncCalc* offers an out-of-the-box solution to aid in CVD detection in NSCLC patients based on routinely acquired imaging data. Comparing to previous works [10–13], *OncCalc* is developed and validated for NSCLC patients and integrates automated CAC assessment with risk stratification to support its clinical utility. It can serve as the gate-keeping step in a two-step CVD risk triage system, to detect patients who would benefit from dedicated cardiac clinic assessment whereby significant past medical history, serum lipid profile, smoking status, hypertension status would be established, thus enabling appropriate subsequent lifestyle and pharmacological management; all whilst incurring no additional radiation exposure to the patient or workload to the imaging department.

The current standard of care which involves visual estimation of CAC burden by the reporting radiologist [7], a study has shown that visual estimation of CAC has a high agreement with manually calculated CAC scores [26], however this study was carried out by only six radiologists highly experienced in reading standard CAC scores prior to the study [26]. Additionally, the relationship between the subspecialty of the radiologist and the diagnostic accuracy of visually estimated CAC has not been evaluated [27]. With a sensitivity of 92% for CAC ≥ 100, OncCalc can act as a first-pass screening for patients with CAC and appropriately refer them for cardiac clinic assessment. This provides the eligible patients with a chance to receive a dedicated follow-up assessment which would include an in-clinic assessment +/- a dedicated calcium scoring scan, with subsequent appropriate CVD risk management.

OncCalc provides a numerical score rather than a crude categorical grading of CAC (mild, moderate or severe), which is arguably a more objective and reproducible measure of disease burden. A quantitative score allows finer CVD risk stratification and may better capture more subtle differences that categorical grading can miss. A limitation of this approach is that non–ECG-gated studies have motion artefacts and blurring related to cardiac motion and patient breathing, restricting the reliability of tracking OncCalc-derived scores longitudinally. Nonetheless, OncCalc remains non-inferior to currently used visual grading system that is currently in use in radiological practice. Additionally, the variation in coronary calcium measurements across serial non–ECG-gated scans may reflect not only true biological progression but also acquisition-related inconsistency, potentially impacting on test sensitivity over time. In a comparative study of AI-based coronary artery calcium scoring on non–ECG-gated chest CT versus ECG-gated cardiac CT, non–ECG-gated chest CT exhibited a 7.5-fold higher risk of misclassification (41.4% vs. 5.5%) and higher false-positive and false-negative rates, highlighting greater variability and potential impact on risk categories when ECG gating is omitted [28]. The definitive gold standard for coronary calcium assessment remains a dedicated, ECG-gated calcium scoring scan, which can be requested following cardiology clinic assessment, if appropriate. As such, CAC assessment on non–ECG-gated imaging should be interpreted as a risk enrichment or triage tool to identify patients who may benefit from dedicated cardiac evaluation, rather than as a substitute for formal ECG-gated calcium scoring.

The variation in model performance observed across external cohorts likely reflects a combination of dataset-specific factors, including scanner vendor, acquisition protocol, slice thickness, image noise, and patient demographics such as CAC burden, age, comorbidities, and smoking history. For example, low-dose or non-contrast CT scans may reduce the visibility of small calcifications, lowering test sensitivity, whereas datasets with a higher prevalence of extensive CAC may yield relatively higher specificity. In the ICHNT cohort specifically, the notably lower specificity is likely also driven by a combination of these characteristics such as variations in scanner protocols or image quality that reduce the model’s ability to distinguish CAC from surrounding structures. Taken together, these factors highlight the importance of multi-centre validation and potential model calibration to account for differences in population characteristics and imaging technique.

While OncCalc is designed to maximise sensitivity to ensure that NSCLC patients with significant CAC are identified, we acknowledge that low specificity may lead to unnecessary cardiology referrals, additional testing, and increased resource utilization. This reflects an expected trade-off, as the model is intended as a first-pass, opportunistic screening tool rather than a definitive diagnostic test. In clinical practice, positive findings from OncCalc would prompt further CVD evaluation including traditional disease risk factors, allowing clinicians to triage patients appropriately. Future work should focus on threshold optimisation, integration with established clinical risk scores, and prospective evaluation of downstream outcomes to balance high-risk patient detection with minimisation of false positives and added healthcare burden.

**Fig. 5:**
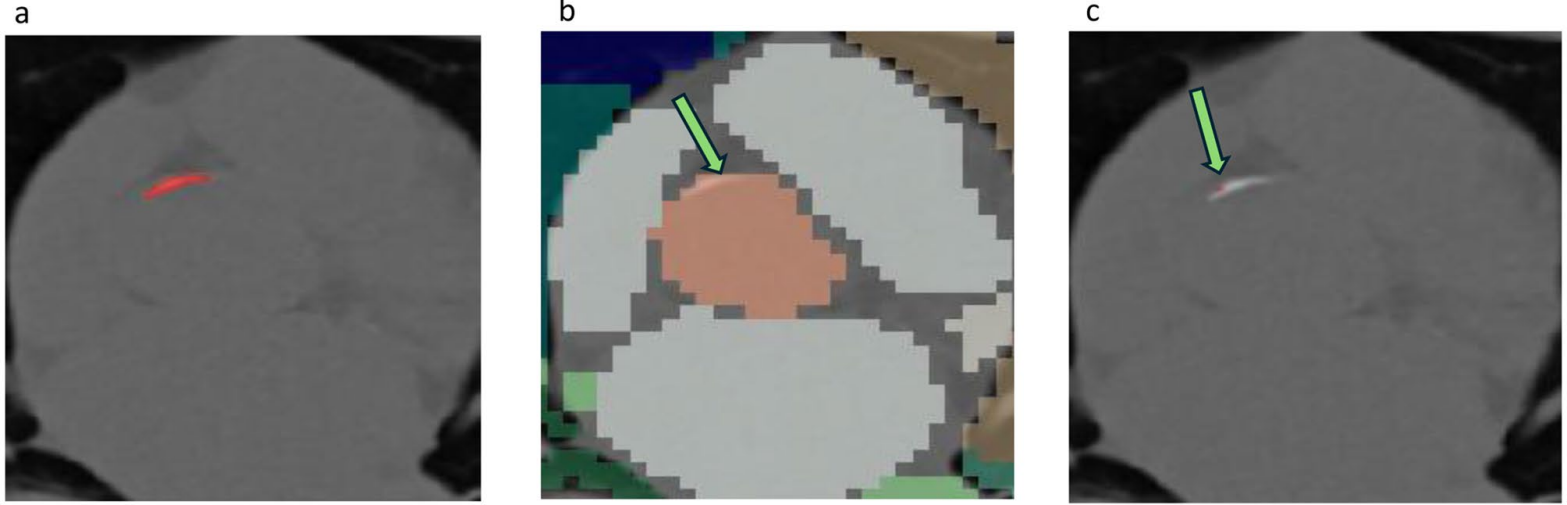
A case without CAC but with ascending aorta calcification. **a**) Ascending aorta calcification incorrectly identified as CAC **b**) TotalSegmentator segments the ascending aorta (green arrow). **c**) The incorrectly identified area in the ascending aorta mostly excluded by the TotalSegmentator-derived mask

Although the raw detection sensitivity of *OncCalc* for significant coronary calcification is 92% for a CAC threshold of 100, its specificity is relatively low at 71%. This can be attributed to multiple phenomena, including the model segmenting non-coronary vascular calcifications, a limitation improved by the integration of TotalSegmentator into the workflow, to incorrectly detected non-CAC calcifications (Fig. 5). On inspection of the ICHNT cohort, there are five images with a negative (< 100) CAC score, however all images are predicted as high risk since these images include abdominal and pelvic regions with calcification which are detected by the model. These false positive cases are resolved by removing the abdomen/pelvis regions of these scans. Further, this disparity in performance across these cohorts may imply a difference in performance across CT protocols which is not generalised by the model.

Automated measurements, especially CAC quantification, are sensitive to acquisition differences. Without domain adaptation or protocol-specific calibration, accuracy may decline when applied outside the original training conditions, as evidenced in our findings. This reinforces the need for multi-centre validation, protocol-aware calibration, and threshold optimisation to ensure robust and clinically meaningful performance across diverse populations and imaging conditions.

In these cases, a false positive classification due to aortic calcification may be helpful rather than harmful. A meta-analysis has shown vascular calcification including that in the coronaries or aorta confers a 3-to-4-fold increase in CVD-related mortality, and coronary events and cerebrovascular accidents (CVA) over a 10-year period [29]. Additionally, a cross-sectional study investigating the relationship between aortic and coronary artery calcifications reported a statistically significant association between the two [30]. The study reports significant coronary calcification is associated with ascending aorta, aortic arch and descending aorta calcification with an odds ratio (OR) of 7.63, 8.46 and 8.73 respectively, and OR of 4.21, 1.65 and 2.14, respectively, when adjusted for CVD risk factors such as body mass index, hypertension and smoking status [30]. Therefore, the detection of aortic calcification by *OncCalc* would still suggest a heightened CVD risk in such patients. Given the important role of statin for the primary prevention of CVD manifestations including the rupture of unstable soft plaques, *OncCalc* can aid on this front through its detection of aortic calcifications, to prompt for dedicated coronary imaging. On the other hand, the mis-indentification of aortic calcification as CAC could contribute to unnecessary referrals where the CAC score is incorrectly calculated to be high risk. Notwithstanding, in this cohort of incorrectly allocated patients, the risk of CVD in the presence of aortic calcification remains significantly greater than the general population, thus these patients may still benefit from a clinical assessment for their CVD risk [29].

As discussed previously, many AI models have been developed and validated for the automatic detection of CAC [10, 11, 31]. A number of these models were developed to automate CAC score calculation on ECG-gated scans to improve efficiency as manual CAC calculation is time-consuming [10, 11, 31]. The previous models developed for low-dose ECG ungated-CT images were mostly developed and validated using different cohort of images and methodologies compared to ours [31–36]. For instance, all other known works used CT scans from databases of the national lung cancer screening and chronic obstructive pulmonary disease (COPD) compared to our dedicated cohort of NSCLC patients [31–36]. Furthermore, methodologies ranged from the training of two convolutional neural networks (CNN) to identify voxels based on their locations and a second CNN to identify calcification according to output of the first CNN to statistical pattern matching approach to automatically calculating CAC [32–34]. In addition, another deep learning-based method included the generation of a coronary map which is fed to the model alongside the CT images to calculate CAC without interference of calcifications outside of the coronary arteries [36].

While attenuation-correction CT from [¹⁸F]FDG PET/CT scans offer a readily available opportunity for opportunistic CAC assessment, they are inherently low-dose, non-ECG-gated, and not optimised for coronary artery evaluation. As a result, these images are more susceptible to increased noise, cardiac motion artefact, and potential inaccuracies in CAC quantification compared with dedicated ECG-gated cardiac CT. These technical constraints necessitate careful validation and limit the direct applicability of CAC detection methods developed for gated cardiac imaging.

To the best of our knowledge, OncCalc is the first dedicated CAC detection model for NSCLC patients based on the state-of-the-art nnU-Net framework. Other models relied on multiple CNNs with bespoke optimisation for coronary artery segmentation and CAC calculation, whereas our method uses a self-configuring, multi-modality approach which configures a single U-net pipeline using the modality and characteristics of the dataset to perform segmentation [18, 31, 32, 34–36]. This makes OncCalc simpler to train, requiring no manual designing of a tailored solution thus making it more scalable and straightforward to fine-tune [18].

Our model’s kappa coefficient reflects a moderate agreement between the model and manual segmentations, whereas an almost perfect agreement was reported in a related work [10]. Notably, our F1-scores are comparable to those reported in that work [10], so despite its moderate agreement, our model’s F1-score of 0.87/0.84, AUC of 0.899/0.926 and a sensitivity of 90.9%/91.9% support the role of the model as an automated opportunistic first-pass CVD risk assessment triaging tool in a context where images would otherwise be of no further clinical value apart from lung cancer staging/screening and patients with high risk of CVD would not be followed up appropriately for CVD risk management.

Although CAC scores from gated and ungated scans show an almost perfect agreement with a kappa coefficient of 0.9 [9], the systematic difference between scores from these two protocols still remains a fundamental limitation of this work, as ungated scans generally yield higher scores than the gated scans [9]. Another limitation of OncCalc is a possible lack of generalisation to different CT protocols as the protocols used to generate the images in the training set are not published so the protocol agnostic status of the model is not well-defined [15]. Nevertheless, one can argue that the potential to save lives with OncCalc as a CVD screening tool in this population is greater than the impact of referring false positive cases.

Importantly, while CAC is a well-established independent predictor of adverse cardiovascular outcomes, direct evidence linking opportunistic CAC detection on oncologic CT to real-world reductions in major adverse cardiovascular events remains limited. Future prospective studies are therefore warranted to evaluate the downstream clinical impact of OncCalc-guided referrals on cardiovascular management decisions and patient outcomes in the NSCLC population.

Looking ahead, with the introduction of lung screening programmes in various countries, this model can be particularly suited to the lung screening population: patients with significant smoking history have elevated risks of both lung cancer and CVD [37–40]. The low-dose unenhanced non-ECG-gated CT chest they receive as part of the screening programme offers a prime opportunity to safety-net patients who with high CAC burdens (> 100) but with clinical risk factors that translate to a borderline or intermediate risk for CVD. Whilst OncCalc is not yet validated for this cohort, it could potentially see use subject to satisfacotry validation findings.

It is important, however, to distinguish between screening and diagnostic workflows when considering opportunistic CAC assessment. In lung screening, low-dose CT scans are primarily acquired to detect early malignancy in high-risk populations, and CAC evaluation is opportunistic, leveraging images that would otherwise not contribute to cardiovascular risk assessment. In contrast, diagnostic workflows, such as staging [¹⁸F]FDG PET/CT in NSCLC patients, are performed for clinical decision-making regarding cancer management, but the same scans can also provide information on CAC without additional imaging or radiation exposure.

Recent literature has examined AI utilisation in low-dose CT lung cancer screening, with findings highlighting general principles equally relevant to opportunistic CAC assessment. As with OncCalc, AI has the potential to increase detection efficiency, standardise interpretation, and identify clinically relevant findings that might otherwise be overlooked [41, 42]. 

## Conclusions

 OncCalc has shown potential to opportunistically screen for CAC on routinely acquired imaging studies with a scope to benefit NSCLC patients. It is designed to complement, rather than to replace radiologists’ assessment of CAC or the need for dedicated calcium scoring scans. It aims to leverage information available on routine CT thorax scans of NSCLC patients who are at a high risk of CVD but could otherwise have missed their diagnoses due to CAC under-reporting, and to prompt for a referral for cardiology clinic assessment, where subsequent lifestyle modification and pharmacological intervention can be considered to reverse/halt the course of CVD progression.

## Supplementary Information

Below is the link to the electronic supplementary material.

Supplementary Material 1

## Data Availability

The study data (clinical and imaging) are retrospective in nature and protected through institutional compliance; they can be shared as per specific institutional review board (IRB) requirements. Upon reasonable request to the first or corresponding author, a data sharing agreement can be initiated between the interested parties and the clinical institution following institution-specific guidelines.

## Code availability

The software and all codes presented in this paper can be found on GitHub: https://github.com/Mitch-Chen-Group/OncCalc.

## Abbreviations

CVD: Cardiovascular disease
CVA: Cerebrovascular accident
NSCLC: Non-small Cell Lung Cancer
CAC: Coronary Artery Calcification
CT: Computed Tomography
FDG PET-CT: F-fluorodeoxyglucose Positron Emission Tomography-Computed Tomography
ICHNT: Imperial College Healthcare NHS Trust
AIMI-COCA: Artificial Intelligence for Medicine
PPV: Positive Predictive Value
NPV: Negative Predictive Value
ECG: Electrocardiogram
BSCI: British Society of Cardiovascular Imaging
BSCCT: British Society of Cardiac Computed Tomography
BSTI: British Society of Thoracic Imaging
AUC: Area Under the Curve
MACE: Major Adverse Cardiovascular Event
TCIA: The Cancer Imaging Archive
ECOG: Eastern Cooperative Oncology Group
ROI: Region of Interest
CPU: Central Processing Unit
RAM: Random Access Memory
VRAM: Video Random Access Memory
ACC/AHA: The American College of Cardiology/American Heart Association
CCS: Canadian Cardiovascular Society
ESC: European Society of Cardiology
CSANZ: Cardiac Society of Australia and New Zealand
CNN: Convoluted Neural Network
ROC: Receiver Operator Characteristic
