# Supplementary material for: Automated opportunistic cardiovascular risk assessment in non-small cell lung cancer patients on routine chest CT using an optimised nnU-net framework
Source: BMC Med Imaging. 2026 Mar 3;26:179. doi: 10.1186/s12880-026-02252-z (PMC13064248; doi:10.1186/s12880-026-02252-z)
Supplement: Supplementary file 1 — Supplementary Material 1 [file 12880_2026_2252_MOESM1_ESM.docx]

**Appendix A: Supplementary Data**


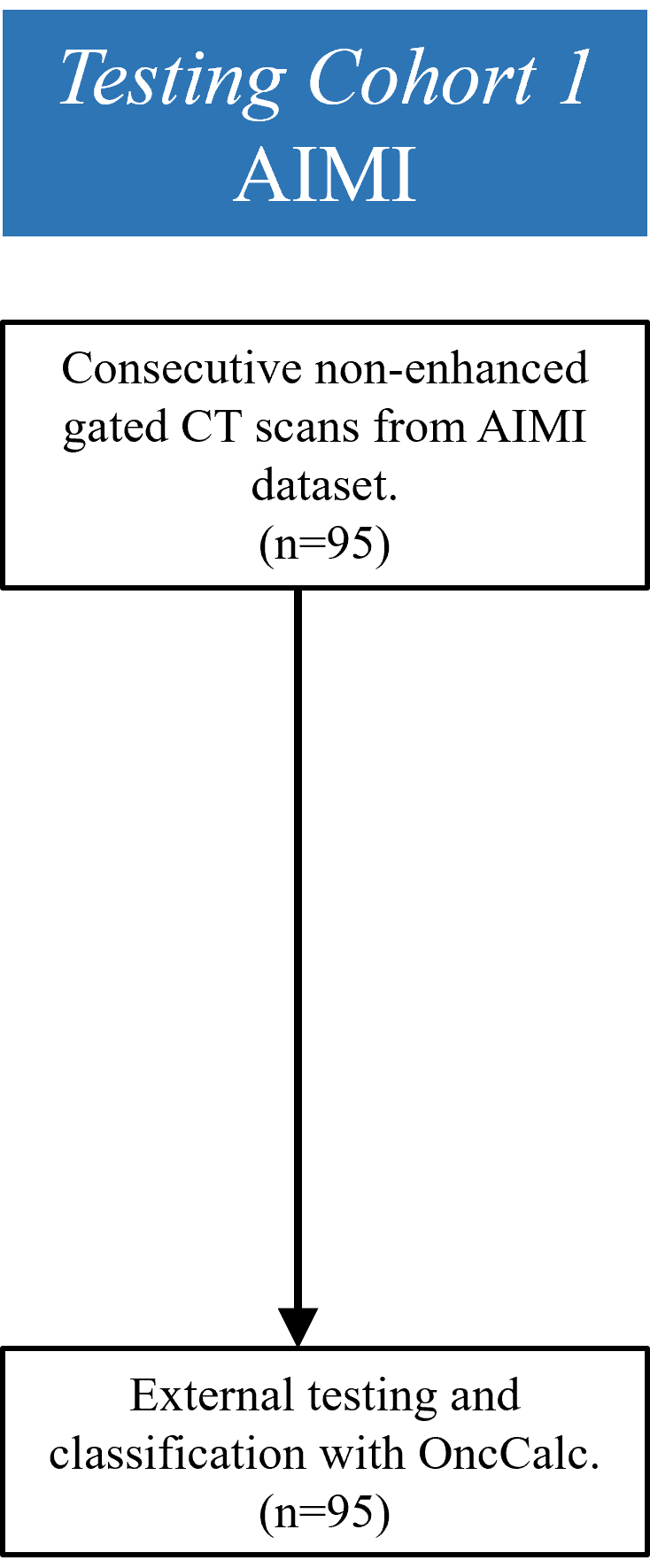

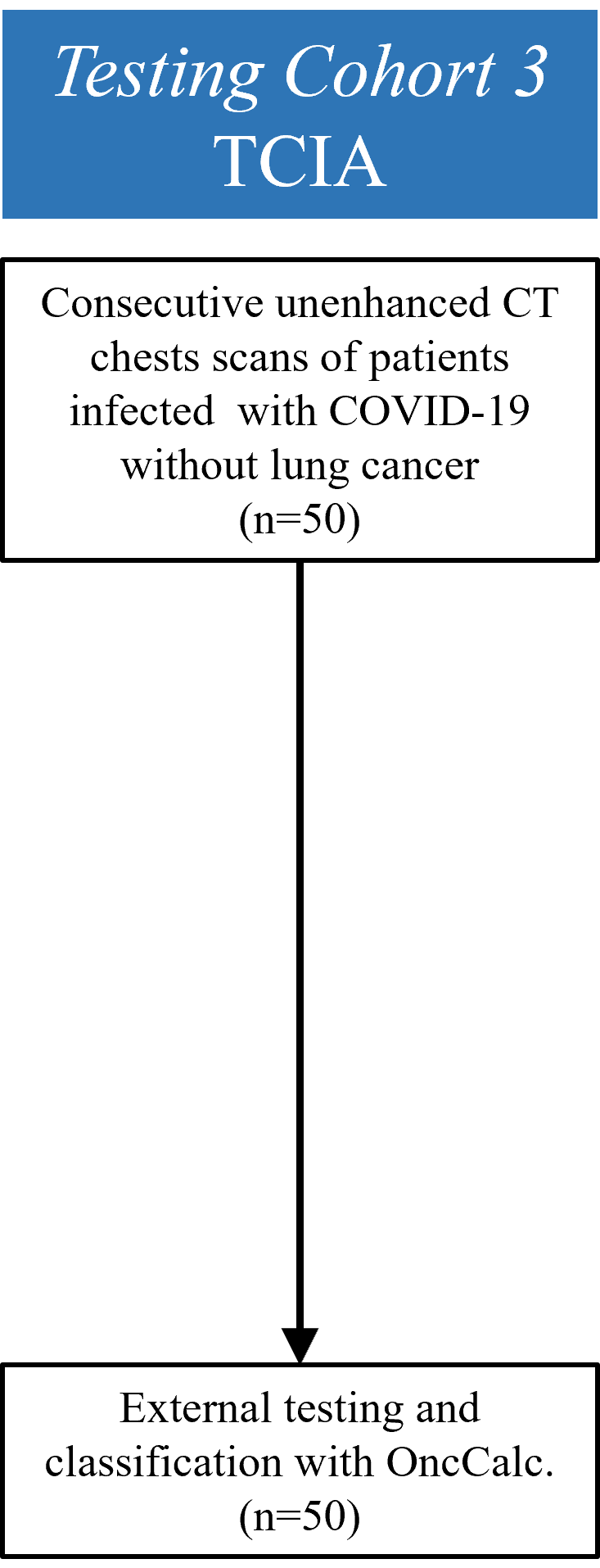

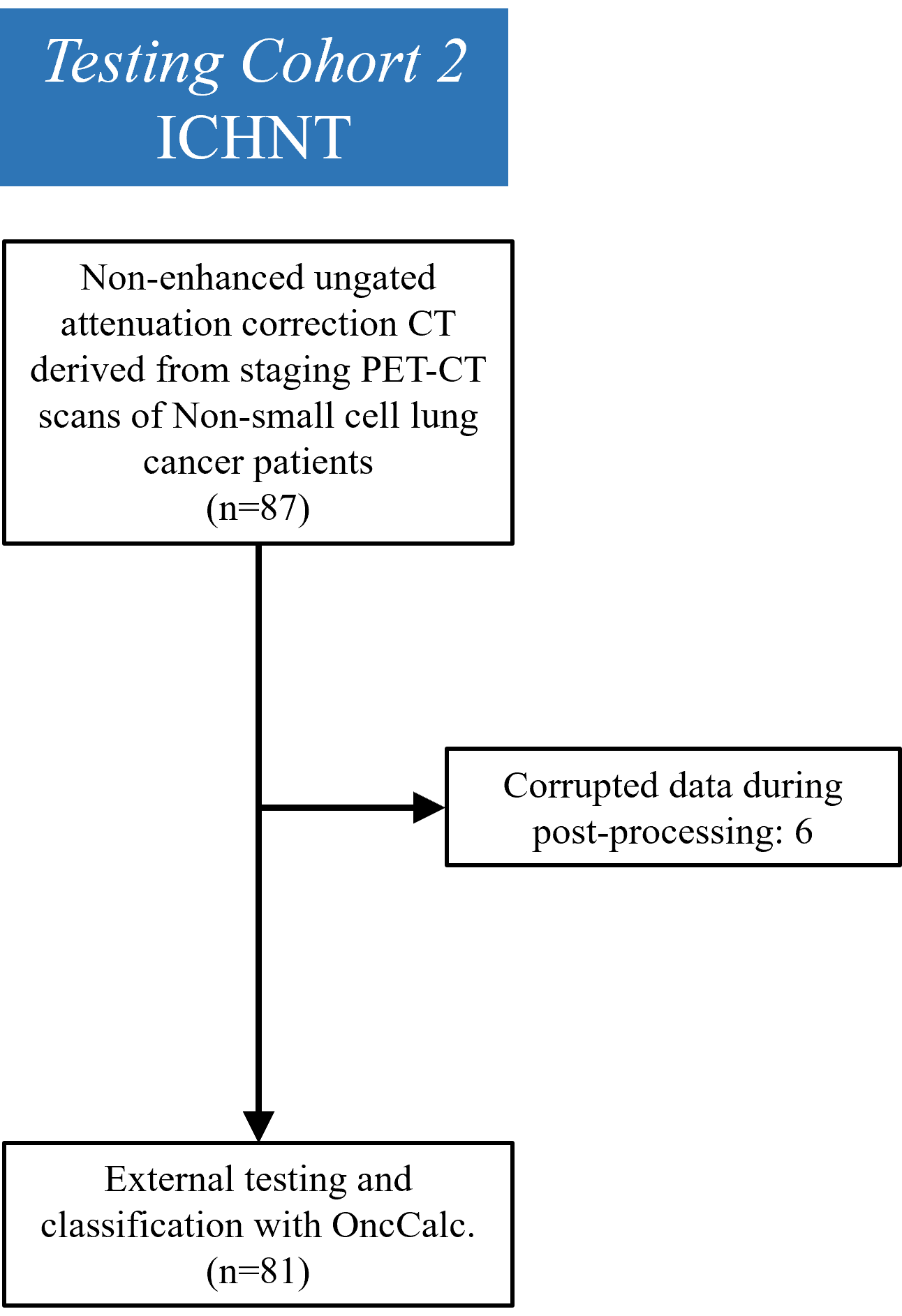


**Figure S1**: CONSORT diagrams of the study cohorts


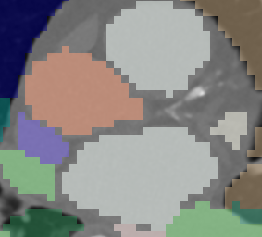

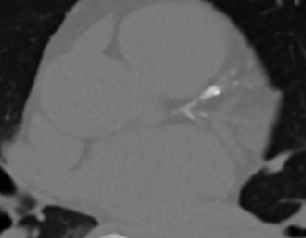

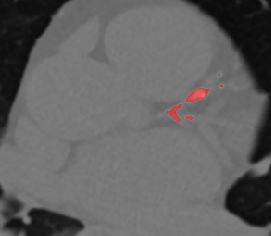


a

b

c

**Figure S2** : O*mitted coronary artery in an TotalSegmentator-analysed case. a) TotalSegmentator-derived mask, the left arrow in this image points to the right ventricle which is coloured in grey (cardiac chambers). The right arrow points to the LAD which has not been segmented b) The original image, with the green arrow pointing to the LAD. c) LAD with CAC highlighted in red. LAD: left ascending descending artery.*
